# Transport of the pituitary adenylate cyclase-activating polypeptide across the blood-brain barrier: implications for migraine

**DOI:** 10.1186/s10194-018-0861-3

**Published:** 2018-05-21

**Authors:** Faisal Mohammad Amin, Henrik Winther Schytz

**Affiliations:** Danish Headache Center, Department of Neurology, Rigshospitalet Glostrup, University of Copenhagen, Valdemar Hansens Vej 1A, 2600 Glostrup, Denmark

**Keywords:** Migraine, PACAP, Blood-brain barrier

## Abstract

**Background:**

Pituitary adenylate cyclase-activating polypeptide (PACAP) is widely distributed in the nervous system and is involved in migraine pathophysiology. Understanding the function of the blood-brain barrier (BBB) in relation to PACAP is important to the understand the mechanisms behind PACAP-induced migraine attacks, but also to develop antimigraine drugs targeting the PACAP receptors Here, we aim to review the transport ability of PACAP across the BBB.

**Methods:**

We performed a systematic literature search on PubMed to identify studies reporting original data on PACAP and BBB. The search was finalized in July 2017.

**Results:**

The literature search identified 96 papers of which 11 contained relevant data. In addition, two papers were known to be relevant and were included. A total of 13 papers studies were included in the final analysis. Preclinical studies (*n* = 10) suggest the existence of specific PACAP *transport* systems across the BBB, while human PACAP studies failed to show vasodilator effect of PACAP on the cerebral arteries from the lumen (*n* = 3).

**Conclusion:**

PACAP38 is transported over the BBB actively, while PACAP27 cross the BBB by diffusion over the membrane, but after crossing the endothelial membrane both isoforms are either rapidly degraded or efflux back from brain to blood. Thus, a direct central action of the PACAPs is unlikely. This is supported by studies showing selective PACAP effect on extra-cerebral arteries.

## Review

The peptide pituitary adenylate cyclase-activating polypeptide (PACAP) is widely distributed in the nervous system and is found in two major isoforms, PACAP38 and PACAP27, which exert various effects via activation of the VPAC1, VPAC2 and PAC1 receptors [1]. A growing body of evidence suggests that PACAP plays an important role in migraine pathogenesis. For example, intravenous infusion of PACAP38 provokes migraine attacks in migraine patients without aura [2, 3] and induces a marked sustained dilation of extra-cerebral but not intracerebral arteries in both healthy volunteers [4] and migraine patients [5]. The exact mechanisms of PACAP38 induced migraine are unknown. However, given the abundant distribution of PACAP in the CNS [6], central pain mechanisms caused by exogenous PACAP infusion could hypothetically be important. This raises the important question to which degree PACAP may cross the blood-brain barrier (BBB). Preclinical studies have reported an active transport system of PACAP38 across the BBB in the rat [7]. However, investigations of rat and human cerebral arteries in vitro, showed that PACAP38 only had effect after abluminal but not luminal application [8]. Understanding the function of the BBB in relation to PACAP is important to the understand the mechanisms behind PACAP-induced migraine attacks triggering, but also to develop antimigraine drugs targeting the PACAP receptors [9]. Here, we aimed to present a systematic review on studies investigating the transport of PACAP across the BBB.

## Methods and materials

We performed a systematic literature search in July 2017 via PubMed to identify studies reporting original data on PACAP in relation to the BBB. The following search terms were used: pituitary adenylate cyclase activating polypeptide or PACAP and blood brain barrier or BBB. No filters were used in the search. Only studies reporting original data published in English-written peer-reviewed journals were included. The first author assessed all titles and abstracts to identify articles containing relevant data. Subsequently, the entire articles were read and additional studies known to be relevant were also included.

## Results

Our search strategy resulted in 96 hits out of which 11 studies were included in the final review. In addition, two studies known to be relevant by the authors were also included ending up with a total of 13 studies (Tables 1 and 2). Two out of 13 studies exclusively examined human arteries [4, 5], while one study reported data on rat and human arteries [8]. The remaining ten studies investigated mice [10–18] or rat models [19]. Only one of the studies were performed as migraine models [5].

**Table 1:** Animal studies investigating PACAP and the blood-brain barrier

Study	Aims	Method	Outcome	Comments
Banks, 1993 [10]	Investigate BBB permeability (influx) for PACAP in the mouse. Efflux from brain to blood was also investigated.	I^125^-labelled PACAP was injected in the jugular vein and blood samples from the contralateral carotid artery after 0.5 to 5 min. Decapitation of the animal immediately after last blood sample. Serum and brain tissue was counted in a gamma counter to determine influx constant Ki. For efflux, I^125^-labelled PACAP was injected in the left ventricle and the brain was removed 2 to 20 min after. Whole brain was counted in a gamma counter.	Influx, Ki for PACAP27 was 2.13 uL/g/min and for 2.86 uL/g/min for PACAP38. Efflux. T1/2 for PACAP27 was 11.8 min and 22.3 min for PACAP38. Unlabelled PACAP38 inhibited influx of labelled PACAP38, which was not true for PACAP27. Unlabelled PACAPs inhibited efflux for their respective counterparts.	0.053% of the iv injected PACAP38 was found in the brain after 5 min or 0.118%iv/g. PTS-6 was suggested to be responsible for active saturable transport across the BBB.
Banks, 1998 [11]	Investigate effect of spinal cord injury on the BBB permeability for PACAP38 and albumin in the mouse.	See Banks, 1993.	Control (healthy) mouse Ki for PACAP38 was 2.42 μL/g/min. In the injuried mouse BBB permeability decreased immediately after injury but increased from day 7 after injury.	I-PACAP was inhibited by unlabeled PACAP. BBB permeability for PACAP was unrelated to the permeability to albumin.
Mizushima, 1999 [12]	Investigate BSCB and BBB permeability for PACAP38 after experimentally induced cardiac arrest in the mouse.	See Banks, 1993. I^131^-PACAP was used instead of I^125^-PACAP.	Reversible increase in BSCB permeability for PACAP38 but no BBB change after cardiac arrest.	The actual BBB permeability values were not reported.
Mizushima, 2000 [13]	Investigate BSCB and BBB permeability for PACAP38 after experimentally induced cardiac arrest in the mouse.	See Banks, 1993. I^131^-PACAP was used instead of I^125^-PACAP.	Reversible increase in BSCB permeability for PACAP38 but no BBB change after cardiac arrest.	The actual BBB permeability values were not reported.
Somogyvári-Vigh, 2000 [19]	Investigate BBB permeability for PACAP38 and albumin after experimental MCA occlusion in the rat.	See Banks, 1993. The penile vein was used for i.v. injection of PACAP instead of the jugular vein.	Ki was 6.5 μL/g/min before and 7.95 μL/g/min after MCA occlusion.	Increased permeability for PACAP38 but not albumin up to 4 h after and normal at 48 h.
Nonaka, 2002 [14]	Investigate BBB permeability for PACAP38 between young and aged mice.	See Banks, 1993. I^131^-PACAP was used instead of I^125^-PACAP.	Young ICR mice: Ki was 7.95 uL/g/min for whole brain. Lowest Ki in the frontal cortex: 3.81 uL/g/min and highest Ki in the hypothalamus: 54.87 uL/g/min and hippocampus: 24.53 uL/g/min. Young SAMP8 mice: Ki was 5.31 uL/g/min for whole brain. Lowest Ki in the thalamus: 3.06 uL/g/min and highest Ki in the hippocampus: 16.96 uL/g/min and hypothalamus: 16.24 uL/g/min. Aged SAMP8 mice: Ki was 2.51 uL/g/min for whole brain. Lowest Ki in the frontal cortex: 2.11 uL/g/min and highest Ki in the hippocampus and hypothalamus: 7.79 uL/g/min.	ANOVA showed difference between young and aged mice. The whole brain difference was only significant between young ICR and aged SAMP8 mice.
Nonaka, 2005 [15]	Investigate BBB permeability (influx) for PACAP38 after intraperitoneal LPS injection in the mouse. Efflux from brain to blood was also investigated.	See Banks, 1993. I^131^-PACAP was used instead of I^125^-PACAP.	PACAP transport rate from blood to brain was not altered by LPS. Efflux. T1/2 for PACAP38 was unchanged 15.6 min after LPS.	Albumin permeability increased after LPS suggesting BBB disruption by the LPS.
Dogrukol-Ak, 2009 [16]	Investigate BBB permeability (efflux) for PACAP27 and PACAP38 in the mouse.	The animal was anesthezised and MCA occlusion was induced. Subsequently, I131-labelled PACAPs with and without a selective antisense targeting the efflux component was injected in the jugular vein. 24 h after MCA occlusion the animal was decapitated and the brain tissue was counted in a gamma counter to determine accumulation of PACAPs in the brain.	PACAP27 but not PACAP38 accumulated in the brain after selective inhibition of the beta-F1 ATPase (efflux component of the PTS-6).	PACAP38 and PACAP27 transport from brain to blood may be via two different mechanisms.
Nonaka, 2012 [17]	Investigate uptake of PACAP38 in the brain after intranasal administration in the mouse.	Intranasal administration of I^131^ labelled and unlabeled PACAP38 and blood sampling from the carotid artery. Decapitation 5–120 min after administration. Serum and brain tissue was counted in a gamma counter to determine	I-PACAP38 uptake occurred in all examined brain regions with the highest uptake in the occipital cortex and striatum (2–4%inj/g). Concomitant administration of unlabeled PACAP38 increased I-PACAP38 in the brain significantly (1000-fold).	Increased amount of I-PACAP38 after concomitant PACAP administration suggests competitive inhibition of the efflux component.
Yu, 2012 [18]	Investigate BBB permeability for PACAP38 with and without combining it with TAT in the mouse.	Recombinant TAT-PACAP38 and PACAP38 were both labeled with FITC and injected i.p. 6 h after injection the animal was decapitated. Brain tissue was centrifuged after flushing blood out of the brain. Determination of FITC-PACAP-TAT and FITC-PACAP was done via fluorimetry.	Uptake of TAT-PACAP38 was 6.55%inj and 2.77%inj for PACAP38.	TAT increased PACAP38s ability to cross the BBB 2.5-fold.

*PTS-6* protein transport system-6, *BBB* blood-brain barrier, *PACAP* pituitary adenylate cyclase-activating polypeptide, *BSCB* blood-spinal cord barrier, *MCA* middle cerebral artery, *ICR* outbred mouse, *SAMP8* a mouse model of Alzheimer, where altered BBB to other substances is known, *LPS* lipopolysaccharide (used to disrupt the BBB), *TAT* a peptide derived from the human immunodeficiency virus 1, *MMA* middle meningeal artery

**Table 2:** Human studies presenting data relevant for understanding PACAP transport across the blood-brain barrier

Grände, 2013 [8]	Investigate effect of PACAP27 and PACAP38 on human and rat arteries.	In vitro myograph of human MCA and in vitro pressurized arteriography of rat MCA.	PACAP38 and PACAP27 had effect after abluminal but not luminal application in the rat. Both PACAPs had effect on the human MCA abluminal.	Luminal application of PACAP to the human MCA was not done in this study.
Amin, 2012 [4]	Investigate effect of i.v. PACAP38 infusion on MCA and MMA in healthy volunteers.	MR-angiography before and after iv infusion of PACAP38.	Large dilation of the MMA but no change of the MCA caliber.	MMA dilation was long-lasting (110 min), while MCA remained unchanged.
Amin, 2014 [5]	Investigate effect of i.v. PACAP38 infusion on MCA and MMA in migraine patients.	MR-angiography before and after iv infusion of PACAP38.	Large dilation of the MMA but no change of the MCA caliber.	MMA dilation was long-lasting (120 min), while MCA remained unchanged. Median time from infusion start to onset of migraine was 4.5 h.

*PTS-6* protein transport system-6, *BBB* blood-brain barrier, *PACAP* pituitary adenylate cyclase-activating polypeptide, *BSCB* blood-spinal cord barrier, *MCA* middle cerebral artery, *ICR* outbred mouse, *SAMP8* a mouse model of Alzheimer, where altered BBB to other substances is known, *LPS* lipopolysaccharide (used to disrupt the BBB), *TAT* a peptide derived from the human immunodeficiency virus 1, *MMA* middle meningeal artery

## Discussion

The main finding in this review is that PACAP can cross the BBB in both directions (i.e. from blood to brain and from brain to blood). Preclinical studies carried out in mice or rats consistently report that PACAP38 is transported actively from the blood to the brain (influx) and from the brain to blood (efflux) by the protein transport system-6 (PTS-6), which is located in the endothelium. In contrast, studies investigating effect of PACAP38 on the cerebral arteries (i.e. middle cerebral artery [MCA]) suggest that PACAP38 is not able to cross the endothelium in sufficient amount to activate receptors in the smooth muscle cells in the arterial walls [4, 5, 8], which will be discussed in the following. Historically, the BBB was considered as a structural barrier between the blood and brain consisting of capillary endothelial cells glued together with tight junctions and surrounded by glial cell projections. We now know that the BBB is a functional rather than just a solid structural barrier, allowing passage in from blood to brain and from brain to blood via different mechanisms. Peptides that cross the BBB are either actively transported by saturable carrier- or receptor-mediated mechanisms or by non-saturable transmembrane or intercellular diffusion. Banks and colleagues [10] first demonstrated that both PACAP27 and PACAP38 could cross the BBB. Interestingly, the permeability for PACAP38 decreased as the concentration of PACAP38 increased in the blood, which was not true for PACAP27. Based on these initial observations authors suggested that PACAP38 traversed the BBB via a saturable mechanism, whereas the PACAP27 uptake must be via a non-saturable mechanism [10]. Ki (influx constant) for PACAP38 (2.86 μL/g/min) was slightly higher than and PACAP27 (2.13 μL/g/min) although PACAP38 is a larger and less lipid soluble molecule. However, the percentage of intact iodine-labelled PACAP27 (I-PACAP27) in the brain was 76.9% versus 58.9% for the PACAP38 [10]. Taken together, these observations suggest efflux of PACAP27 from the brain to blood via the saturable beta-F1 ATPase, which is highly specific for PACAP27 [16]. Interestingly, the passage of PACAP38 was not affected by a general disruption of the BBB by lipopolysaccharide [15], but after occlusion of the MCA [19].

Two human MR-angiography studies found no functional effect of intravenously infused PACAP38 on the MCA but a 17–19% and 2 h sustained dilation of the middle meningeal artery (MMA) in healthy volunteers [4] and migraine patients [5]. Interestingly in vitro studies reported presence of PACAP receptors in the meningeal as well as cerebral arteries [20, 21]. Thus, the selective dilation of the MMA but not the MCA may suggest that intravenously administrated PACAP38 most likely did not cross the BBB. Another human study has shown that infusion of PACAP38 can induce premonitory symptoms in 48% of patients with induced attacks [22], which may be a central effect via the hypothalamus [23]. However, PACAP38 infusion did not induce more premonitory symptoms in patients who developed an attack compared with those who did not develop an attack, which suggest that induction of premonitory symptoms is not a necessary mechanism for the induction of migraine. Moreover, PACAP38-induced migraine attacks are accompanied by change in the intrinsic brain connectivity [24]. These observations suggest either that there is a difference between species or that the efflux mechanisms for PACAP38 work even faster in man to transport PACAP38 from the endothelial cells back to the blood. Thus, PACAP cannot exert its effect although it can cross the BBB or enter the endothelial cells. In contrast, Nonaka et al. [14] reported the highest amount of iodine-labelled PACAP38 (I-PACAP38) in for instance the hypothalamus. Alternatively, the PTS-6 system may be limited to the capillary endothelium and not the large arteries of the brain, where the endothelium may work as a structural barrier denying access to PACAP38 from the blood to the smooth muscle cell where the PACAP-receptors are located. PACAP-receptors are found in the entire brain and one study reported uptake of I-PACAP38 in all brain regions after intranasal administration [17]. It would be interesting to directly compare effect on the central nervous system and cerebral arteries after intravenous and intranasal administration.

It has previously been speculated and suggested that the BBB might be more permeable during migraine attacks [25]. However, recent advanced MRI using intravenous gadolinium contrast performed during and outside of attacks of migraine with [26] and without aura [27] reported no significant change in the BBB permeability. The clinical impact of determining whether intravenous PACAP has a CNS effect is related to the potential of developing PACAP or PACAP receptor antibodies as treatment. Currently, there is an ongoing PAC1 receptor antibody trial for migraine treatment (ClinicalTrials.gov Identifier: NCT03238781).

## Conclusion

PACAP38 transport from the blood to brain and from brain to blood is saturable carrier-mediated, whereas PACAP27 crosses the BBB from the blood to brain by transmembrane diffusion. The transport of PACAP27 the brain to blood is mediated by a saturable and peptide-carrier. There is no solid evidence yet of PACAP38 passing the BBB following exogenous infusion in migraine models that can lead to brain function and brain vessel changes, so it is possible that the PACAP migraine inducing effect is caused by peripheral mechanisms, which could be via inducing changes in the meninges or affecting nociceptors in extracranial vessels both devoid of the BBB. Future advanced in vivo human studies and exploration of PAC1-receptor antibodies using radio-labelled tracers are highly needed to clarify how and where PACAP may lead to the development of migraine pain.

## Abbreviations

BBB: Blood-brain barrier
I-PACAP27: Iodine-labelled pituitary adenylate cyclase-activating polypeptide-27
MCA: Middle cerebral artery
MMA: Middle meningeal artery
PACAP: Pituitary adenylate cyclase-activating polypeptide
PACAP27: Pituitary adenylate cyclase-activating polypeptide-27
PACAP38: Pituitary adenylate cyclase-activating polypeptide-38
PTS-6: Protein transport system-6
